# Association of Adiponectin Polymorphism with Metabolic Syndrome Risk and Adiponectin Level with Stroke Risk: A Meta-Analysis

**DOI:** 10.1038/srep31945

**Published:** 2016-08-31

**Authors:** Hui-Ping Yuan, Liang Sun, Xing-Hui Li, Fu-Gang Che, Xiao-Quan Zhu, Fan Yang, Jing Han, Chun-Yuan Jia, Ze Yang

**Affiliations:** 1 The Key Laboratory of Geriatrics, Beijing Hospital, National Center of Gerontology & Beijing Institute of Geriatrics, Beijing 100730, P.R. China

## Abstract

Many previous studies have provided evidence that the ADIPOQ +45T>G polymorphism (rs2241766) might cause metabolic syndrome (MS). As a cardiovascular manifestation of MS, the incidence of stroke is associated with adiponectin; however, the results remain controversial and inconsistent. Systematic searches of relevant studies published up to Dec 2014 and Jan 2016 on the ADIPOQ +45T>G polymorphism and the risk of MS and adiponectin levels and the risk of stroke, respectively, were conducted in MEDLINE and EMBASE. The odds ratio (OR) or risk ratio (RR) and their 95% confidence interval (95% CI) were extracted. Sixteen studies containing 4,113 MS cases and 3,637 healthy controls indicated a weak positive association between ADIPOQ +45 T>G and MS in the dominant genetic model (OR = 1.30, 95% CI = 1.03–1.65), which was also validated by stratified subgroup analyses. Twelve studies including 26,213 participants and 4,246 stroke cases indicated that 5 μg/ml increments in adiponectin level were not relevant to stroke risk (RR = 1.05, 95% CI = 1.00–1.10, P = 0.069). This study suggested a weak positive association of ADIPOQ +45T>G with MS and a strong association with metabolic-related disease. Additionally, adiponectin level was not a causal factor of increasing stroke risk.

Metabolic syndrome (MS) constitutes a group of risk components including abdominal obesity, insulin resistance, hyperglycaemia, hyperlipidaemia, and hypertension^1^. This disorder is currently widely prevalent, present in 20–25% of the world’s adult population, and is particularly common in the industrialized societies of the world, where it is prevalent in epidemic proportions. Both genetic and environmental factors contribute to the development of MS^2^. Many studies have demonstrated the genetic mechanisms of MS and metabolic-related disease, but these mechanisms remain controversial and require further research.

Adiponectin is the most abundant adipose tissue-derived adipocytokine encoded by *ADIPOQ* (also known as *APM1*), which is located on chromosome 3q27, spans 17 kb, contains 3 exons and 2 introns^3^,^4^, and comprises 247 amino acids^5^. It is involved in the regulation of glucose and lipid metabolism, cardiovascular function, and amelioration of insulin resistance (IR) and has anti-inflammatory, anti-diabetic and anti-atherogenic properties; additionally, low circulating levels of adiponectin are associated with central obesity, insulin resistance, MS, and type 2 diabetes (T2DM)^6^,^7^. Great attention has paid to *ADIPOQ*, which is considered an important factor in MS and metabolic-related disease. Therefore, identifying the intrinsic genetic mechanisms and clarifying the similarities as well as partial distinctions of these mechanisms are issues that we pursue with interest in this study.

*ADIPOQ* +45T>G (rs2241766) is one of the most common variants of exon 2 on the *ADIPOQ* gene, and some studies have provided evidence that the *ADIPOQ* +45 T>G polymorphism is associated with serum levels of adiponectin^3^, MS components, insulin sensitivity, obesity and T2DM^8^. To our knowledge, the results to date have been discordant. There has been a meta-analysis conducted by GAO M *et al*. that differed from ours^9^, but that study was limited to the Chinese population, lacked a detailed stratified analysis, and used data that were collected before August 2012. Thus, we conducted an updated analysis on the relationship between *ADIPOQ* +45 T>G and MS risk.

In addition, as a common cardiovascular manifestation of MS, the association between the incidence of stroke and adiponectin has also been inconsistent^10^,^11^,^12^. Although Arregui M *et al*. conducted a meta-analysis including 9 studies on this topic; the overall RR estimate might have been inaccurate due to the significant publication bias and obvious heterogeneity, which both reduced the validity and accuracy of the conclusion.

Considering the lack of knowledge on the specific *ADIPOQ* genetic determinants of MS, the controversy regarding the association between adiponectin and stroke, and the need to update the latest data with stratified subgroup analyses to obtain more accurate conclusions, the objective of the present updated study was as follows: to investigate the latest data among the whole population and obtain greater statistical power to detect the association between the *ADIPOQ* +45 T>G polymorphism and MS; to further assess the relationship between *ADIPOQ* +45 T>G and metabolic-related disease in a published meta-analysis and beyond that, compare the similarities and differences in genetic background between MS and metabolic-related disease; and to examine the relationship between adiponectin and risk of stroke.

## Methods

This study included two meta-analyses. The primary one assessed the relationship of the *ADIPOQ* +45 T>G polymorphism and MS risk, and the secondary one investigated the dose-response relationship of adiponectin and risk of stroke.

### Literature Searches

This study was conducted according to the meta-analysis guidelines defined in the PRISMA statement^13^,^14^. The publication search was carried out in multiple electronic databases: PubMed, EMBASE, CNKI (China National Knowledge Infrastructure), and Wanfang databases.

For the primary analysis of the *ADIPOQ* +45 T>G polymorphism and MS risk, the following subject terms were used to conduct the search: “adiponectin” or “*ADIPOQ*” or “adiponectin gene polymorphism”, “*AMP1*”, and “metabolic syndrome” or “MS” or “metabolic syndrome X” or “syndrome X”. The relevant articles in English and Chinese published before Dec 31, 2014, were included.

For the secondary subject of adiponectin and stroke risk, the following subject terms were used for the search: “adiponectin” and “stroke”. The relevant articles in English published before Jan 26^th^, 2016, were included.

We also conducted a manual search to find additional relevant data based on the references identified in the retrieved articles.

### Inclusion and Exclusion Criteria

The inclusion criteria of the primary meta-analysis were as follows: the studies (1) evaluated the relationship between *ADIPOQ* +45T>G (rs2241766) and MS risk; (2) were case-control studies; (3) provided detailed genotype data to estimate odds ratios (ORs) and 95% confidence intervals (CIs).

The inclusion criteria of the secondary meta-analysis were as follows: the studies (1) used a prospective cohort to investigate the relationship of adiponectin and stroke risk; (2) included stroke as an endpoint; (3) had a follow-up duration of over 1 year; (4) used clear diagnostic criteria for stroke; and (5) provided data on the relative risk (RR) with 95% CI. In all identified studies, all subjects were free of stroke at baseline according to the stroke diagnostic criteria used and participated in the entire follow-up survey.

All the articles that met the above requirements were included, regardless of the sample size and the population of the studies. Unpublished conference papers were not included. For overlapping studies, the most recent publication or the largest sample size publication providing more information was selected.

### Data Extraction

Two investigators (H.-P.Y. and L.S.) systematically extracted and reviewed all studies according to a standardized form and then independently entered this information into an electronic database. The following items were reviewed and extracted from each eligible study for the primary meta-analysis when possible: name of the first author, year of publication, ethnicity, country where the study conducted, mean age, total number of cases and controls, allele frequencies, genotyping method, diagnostic criteria of MS, and language of the article. Different ethnicity descents were categorized as East Asian, South Asian, and Southeast Asians.

The extracted information for the articles for the secondary meta-analysis included the name of the first author, publication year, name of the study, country, mean age, duration of follow-up, proportion of men, number of stroke cases and total participants, and adjusted variables. In addition, all of the multivariate adjusted RRs and 95% CIs based on a comparison of the highest adiponectin level category with the lowest adiponectin level category and the ORs/RRs with their 95% CIs were extracted. We abstracted the values for each category of adiponectin level, the median value of the adiponectin level for each category, the number of cases and participants/person-time for each category, and the most completely adjusted RRs with 95% CIs (odds, hazard or risk ratios were combined into RRs) for each category.

### Quality assessment

A quality assessment of the primary meta-analysis was performed by two authors independently according to the Newcastle-Ottawa scale^15^ for non-randomized studies. The scale contains nine points: selection (4 points), comparability (2 points), and estimation of outcomes or exposures (3 points).

The qualities of the identified studies for the secondary meta-analysis were assessed by two authors independently according to a “methodological quality assessment scale” (Supplementary Table S3), which was modified from a previous publication^16^,^17^. Five items including the representativeness of the cases, source of controls, sample size, quality control of the genotyping methods, and Hardy–Weinberg equilibrium (HWE) were assessed in this scale. The quality score ranged from 0 to 10, with a high score indicating a good quality study. Any disagreements between the two investigators were resolved through discussion.

### Statistical Analysis

The primary meta-analysis of the relationship between the *ADIPOQ* +45 T>G polymorphism and MS risk was conducted as follows. ORs and 95% CIs were calculated as estimates of the relative strength of the associations between the *ADIPOQ* +45 T>G polymorphism and MS risk. The pooled ORs were calculated from a weighted average of OR from each study. Due to the potential functional role of the *ADIPOQ* +45 T>G polymorphism, carriers with at least one risk allele will present the corresponding phenotype or traits. Moreover, to obtain all of the data included, the dominant model (GG + GT *vs.* TT) was adopted. Allele contrast (G *vs.* T) and recessive models (GG *vs.* GT + TT) were also performed in the meantime. After adjusting for the potential confounding factors that might have contributed to the heterogeneity, the dominant model was adopted for further investigation. Subgroup analyses were also conducted to explore the effects of the following confounding factors: language of the article, diagnostic criteria, and ethnicities.

The secondary meta-analysis of the relationship between adiponectin and risk of stroke was conducted as follows. Multivariable-adjusted RRs and 95% CIs were used to obtain the overall RR for the highest adiponectin level category compared with the lowest category using random-effects models. RRs and 95% CIs for each SD increase in log-transformed adiponectin were used to obtain the overall RR per SD in log μg/ml. The RR of each 5 μg/ml adiponectin level increment was calculated for studies that provided enough raw data to quantify the association between adiponectin level and risk of stroke using GLST (generalized least-squares trend estimation) and VWLS (variance-weighted least square regression model) for the dose-response analysis, as proposed by Greenland, Longnecker, Orsini *et al*. The detailed methods of the dose-response meta-analysis have been described before^18^.

The between-study heterogeneity was calculated using Cochran’s Q test (Mantel-Haenszel chi-squared test) and was considered significant if the *P* value was less than 0.1^19^,^20^. Additionally, the *I*^2^ statistic was used to test for heterogeneity, with values of *I*^2^ less than 25%, 25–50%, and greater than 50% representing low, moderate and high degrees of inconsistency, respectively. When the *P* value was ≥0.1 or *I*^2^ ≤ 25%, a fixed effects model (Mantel–Haenszel method) was selected; otherwise, a random effects model (DerSimonian and Laird method) was selected. We then explored the sources of heterogeneity using meta-regression and stratified analysis. Sensitivity analyses were performed to evaluate the stability of the results. A leave-one-out method was used to evaluate each study, and a pooled estimate was calculated for the remaining studies. Begg’s funnel plots were conducted to evaluate the publication bias qualitatively; Begg’s test and Egger’s test were performed to quantitatively assess the publication bias. All *P* values were two-sided, with the statistically significant level defined as lower than 0.05. The statistical analyses were conducted using Stata (version 12.0; StataCorp LP, TX, USA).

## Results

### Characteristics of the Included Studies

The characteristics of each study included in these two meta-analyses are summarized in Tables 1 and 2.

Supplementary Fig. S1A shows the detailed procedure by which articles are identified and assessed for inclusion in the primary meta-analysis. Table 1 illustrates the characteristics of all the included studies in this first meta-analysis. The 16 studies^9^,^21^,^22^,^23^,^24^,^25^,^26^,^27^,^28^,^29^,^30^,^31^,^32^,^33^,^34^,^35^ contained 4,113 MS cases and 3,637 healthy controls. The distributions of the genotype frequencies of the controls were all consistent with the HWE (all *P* > 0.05). The serum adiponectin levels presented in Table 1 shows that the concentration of adiponectin is lower in MS patients and higher in controls (*P* = 0.0016).

Supplementary Fig. S1B shows the detailed procedure by which articles are identified and reviewed for inclusion in the secondary meta-analysis. The 12 studies^11^,^36^,^37^,^38^,^39^,^40^,^41^,^42^,^43^,^44^,^45^,^46^ in this analysis contained 26,213 participants and 4,246 stroke cases. Seven studies^11^,^36^,^37^,^40^,^41^,^43^,^44^ (8 data points due to separate data for men and women) were included to compare the highest adiponectin level category with the lowest category. Two studies^37^,^45^ (3 data points due to separate data for men and women) were included to estimate the RR per SD increase in log-transformed adiponectin level. Finally, 11 studies^11^,^36^,^37^,^38^,^39^,^40^,^41^,^42^,^43^,^44^,^46^ (13 data points due to separate data for men and women) were included to estimate the dose-response relationship between adiponectin level and stroke.

### Quality assessment

Quality assessments were performed for the included studies both in the primary and the secondary meta-analyses. All studies had acceptable qualities according to the methodological quality assessment (Table 1 and Supplementary Table S3) and the Newcastle-Ottawa scale (Supplementary Table S4).

### ADIPOQ +45 T>G polymorphism and MS risk

**Figure 1:**
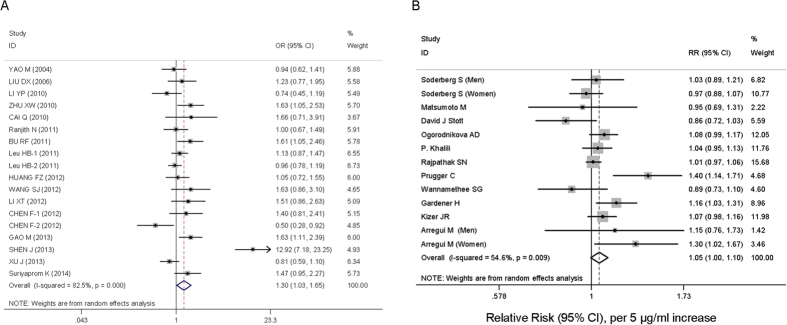
Forest plots of the two meta-analyses. (**A**) The relationship between *ADIPOQ* +*45*T>G and the risk of metabolic syndrome in the dominant model. The odds ratio (OR) and 95% confidence intervals (CIs) are presented graphically by a square box and horizontal line, respectively. Box sizes are proportional to inverse-variance weights. The diamond represents the overall OR with its 95%CI using a random effects model. Leu HB-1 and Leu HB-2 represent two separate populations. CHEN F-1: MS; CHEN F-2: MS with CHD. (**B**) The relationship between adiponectin level and the risk of stroke under a random effects model. The risk ratio (RR) and 95% CIs are presented graphically by a square box and horizontal line, respectively. Box sizes are proportional to inverse-variance weights.

A weak positive association was visible in the relationship between *ADIPOQ* +45 T>G and MS under the dominant genetic model (OR = 1.30, 95% CI = 1.03–1.65) (Fig. 1A). Results of allele contrast and recessive models were consistent with the dominant model.

### Adiponectin levels and stroke risk

The combined RR of stroke was 1.11 (95% CI = 0.87–1.41, *P* = 0.389) for the highest adiponectin level category compared to the lowest adiponectin level category (Supplementary Fig. S2). Two studies (3 points) that provided the RRs of stroke per SD in log-transferred adiponectin level demonstrated that the overall RR was 1.20 (95% CI = 0.99–1.44, *P* = 0.059). Additionally, 11 studies with 13 data points that provided sufficient raw data for the dose-response analysis indicated that the RR of stroke for each 5 μg/ml adiponectin level increment was 1.05 (95% CI = 1.00–1.10, *P* = 0.069) (Table 3 and Fig. 1B). After excluding two papers by Prugger C *et al*. and Gardener H *et al*., the RR of stroke for each 5 μg/ml adiponectin level increment was 1.02 (95% CI = 0.99–1.05, *P* = 0.261; *I*^2^ = 27.8%). Therefore, there was no association between adiponectin levels and stroke risk.

### Stratified Analysis and Meta-Regression

Considering the significant heterogeneity in both meta-analyses, a stratified analysis and meta-regression were performed successively to investigate the potential sources of heterogeneity.

For the primary meta-analysis, first of all, we conducted a stratified analysis based on language and diagnostic criteria of the overall data and found that they were not the sources of heterogeneity (*I*^2^ was largely unchanged, data were shown in Supplementary Fig. S3A,B). In addition, the results of the sensitivity analysis and publication bias analysis showed that the data from SHEN J *et al*. and CHEN F-2 *et al*. deviated substantially from most studies. After removal of the two studies, a weak positive association was still visible in the relationship between *ADIPOQ* +45 T>G and MS under the dominant genetic model (OR = 1.13, 95% CI = 1.03–1.24). Furthermore, *I*^2^ was reduced from 82.5% to 40.1%, with *P = *0.050 (Supplementary Fig. S3A,B). Based on these findings, as well as similar results observed in the meta-regression (Tau-square significantly reduced), we excluded the SHEN J *et al*. and CHEN F-2 *et al*. studies from further analysis. In the subsequent stratified analysis based on language, we arrived at the conclusion that under the dominant model, a weak positive association was found in the relationship between the +45T>G polymorphism and MS susceptibility (in Chinese, OR: 1.12, 95% CI: 0.97–1.28; in English, OR: 1.14, 95% CI: 1.00–1.30) (Supplementary Fig. S3A). Further stratified analyses based on the diagnostic criteria suggested the same results (WHO: OR = 1.10, 95%CI = 0.78–1.55; IDF: OR = 1.05, 95%CI = 0.89–1.25; CDS: OR = 1.55, 95%CI = 1.22–1.97; NCEP ATP III: OR = 1.07, 95%CI = 0.92–1.23) (Supplementary Fig. S3B). As we can see from Supplementary Fig. S3A, data from the studies in English and from those that used the CDS diagnostic criteria seems to be more consistent (English: *I*^2^ = 40.8%, *P* = 0.134; CDS: I^2^ = 0.1%, *P* = 0.974). Finally, considering the differences in the genetic background of the population and that the risk allele frequency of +45 T>G polymorphism differed greatly among ethnicities, we further performed a subgroup analysis on East Asians. Asian Indians (South Asians) and individuals from Thailand (Southeast Asians) were excluded in this analysis. Data from East Asians leads to the same conclusion (OR = 1.17, 95%CI = 1.01–1.35, *P* = 0.035) (Supplementary Fig. S3C).

For the secondary meta-analysis, the stratified analysis was performed based on study design, country, mean age, sex, follow-up duration, and measure of association (data were shown in Supplementary Table S1). There was no relationship between the risk of stroke and elevated adiponectin levels and it remained for all factors we proposed (i.e., all RR values were around 1). We found that these factors were not the sources of heterogeneity (*I*^2^ was largely unchanged). The meta-regression showed that the association did not substantially differ due to the effect of these potential confounding factors (i.e., all *P* were >1).

### Sensitivity Analysis

As shown in Supplementary Fig. S4A, one-way sensitivity analyses for the primary analysis were performed by excluding one study at a time in each genetic model. The results of the dominant genetic models indicated that the overall results were dependable. As we can see, the two papers by SHEN J *et al*. and CHEN F-2 *et al*. were far from the centre line, which might have influenced the overall tendency. For the secondary analysis, two papers by Prugger C *et al*. and Gardener H *et al*. appeared far from the centre line, which might have influenced the overall tendency (Supplementary Fig. S4C). After removing these studies, the tau-squared decreased for the primary and secondary analysis (from 0.1354 to 0.0194 for the primary analysis and from 0.0038 to 0.0011 for the secondary analysis) and explained 85.67% and 71.1% of the sources of heterogeneity, respectively.

### Summary of Meta-Analysis Related to *ADIPOQ* +45T>G

**Figure 2:**
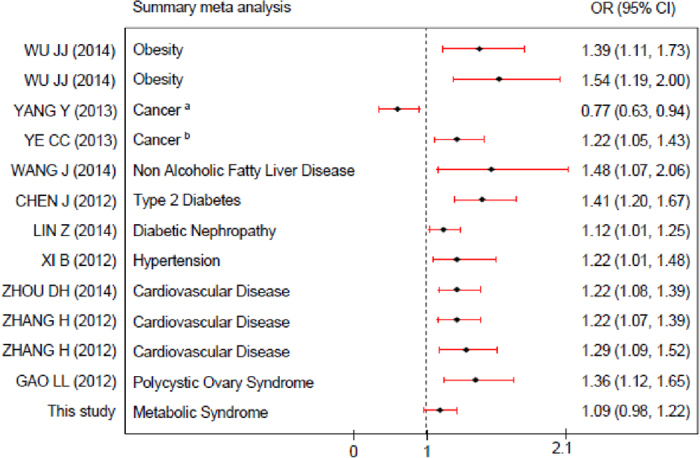
Summary of meta-analysis on the role of ADIPOQ +45T>G in potential metabolic-related disease reported to date. The odds ratio (OR) and 95% confidence intervals (CIs) are presented graphically by a square box and horizontal line, respectively. ^a^cancer; ^b^colorectal cancer.

Considering the close relationship between *ADIPOQ* +45 T > G and the typical components of MS as well as the many resulting potential disease risks such as obesity, cancer, and hypertension, we summarized data from the published meta-analyses as well as the primary meta-analysis in our study (Fig. 2, Supplementary Table S2). The results showed that *ADIPOQ* +45 T>G appeared to be a strong risk factor for MS components, obesity, non-alcoholic fatty liver disease (NAFLD), T2DM, hypertension, cancer, diabetic nephropathy (DN), cardiovascular disease (CAD), coronary heart disease (CHD) and polycystic ovary syndrome (POVS).

### Test of Heterogeneity

For the primary analysis, we performed a heterogeneity test based on the dominant model in our meta-analysis and found strong heterogeneity among the included studies (*I*^2^ = 82.5%, *P* < 0.001) (Fig. 1A). The allele contrast and recessive genetic models also showed substantial heterogeneity (data not shown). It was thus very important to identify the sources of heterogeneity and weaken their influence.

For the secondary analysis, clear heterogeneity was observed for both the RR of the highest adiponectin level category compared with the lowest category and the RR of each 5μg/ml increment in adiponectin level (*I*^2^ = 54.6%, *P* = 0.009) (Fig. 1B and Supplementary Fig. S2).

Due to the high heterogeneity in the two meta-analyses, stratified analyses, meta-regression, and sensitivity analyses were conducted. Based on the results above, we found that although some studies were far from the central line in the sensitivity analyses and might have been the sources of the heterogeneity, the conclusions regarding the associations in the two meta-analyses did not change when these studies were excluded. Therefore, the results of the two meta-analyses were reliable and stable.

### Publication Bias Analysis

As shown in Supplementary Fig. S4B, we found no obvious asymmetry in the dominant genetic model in the primary analysis, and this result was statistically supported by the Begg’s and Egger’s test (*P*_*Begg’s*_ = 0.325, *P*_*Egger’s*_ = 0.118). Additionally, the studies by SHEN J *et al*. and CHEN F-2 *et al*. were found to have deviated far from the others. For the secondary analysis, there was no obvious publication bias based on the funnel plot, Begg’s test, and Egger’s test (*P*_*Begg’s*_ = 0.631, *P*_*Egger’s*_ = 0.378) (Supplementary Fig. S4D).

## Discussion

Adiponectin is a plasma glycoprotein of adipose tissue origin^47^. Adiponectin levels were reported to be lower in subjects with the MS phenotype, including obesity, T2DM, dyslipidemia, and hypertension^48^. Studies also confirmed that the *ADIPOQ* gene (+45T>G polymorphism) is associated with adiponectin levels and resulting MS^9^,^49^,^50^. However, the findings are still conflicting. Therefore, we conducted an updated meta-analysis to derive a more precise evaluation of the association of the *ADIPOQ* +45 T>G polymorphism and MS risk. Furthermore, genetic factors like the *ADIPOQ* +45T>G polymorphism is related to serum adiponectin levels^51^,^52^. Thus, in addition to the *ADIPOQ* +45T>G contribution to MS, adiponectin levels might also contribute to stroke risk, which is considered a cardiovascular manifestation of MS^12^,^53^. Due to the inconsistent conclusions regarding the association between adiponectin levels and stroke risk, 12 studies on this topic were also included in our secondary meta-analysis.

In the primary analysis, due to the strong heterogeneity, a sensitivity analysis, stratified analysis and meta-regression were conducted in the dominant genetic model. Diversity in ethnicity, diagnostic criteria, and distinction between published English and Chinese language studies might be the common source of heterogeneity^54^,^55^,^56^, thus we tried to clarify the sources of heterogeneity through subgroup analysis by the above three factors. The stratified analysis and meta-regression indicated that language and diagnostic criteria were not sources of heterogeneity (Tau-squared value was not decreased). Meta-analysis is often affected by the potential “outlier points” among the involved studies, which might be correlated to the data reliability, small sample size, publication bias and clinical confounding. In the current meta-analysis, both of sensitivity analysis and publication bias analysis indicated 2 articles from SHEN J *et al*. and CHEN F-2 *et al*. deviated potentially from other studies (Supplementary Fig. S4A,B). The articles from SHEN J *et al*. and CHEN F-2 *et al*. contributed inappropriate data to the overall assessment and were thus removed. After their exclusion, the heterogeneity greatly improved (I2 reduced to 40.1%) and the Tau-squared value reduced accordingly whereas this positive association still existed. Therefore, we excluded the 2 studies for the further stratified analysis and meta-regression. Taken together, our study indicated that a weak positive association was visible in the relationship between *ADIPOQ* +45T>G and MS in the dominant genetic model (OR = 1.30, 95% CI = 1.03–1.65).

The first meta-analysis focused on the relationship between the *ADIPOQ* +45T>G polymorphism and the risk of MS in the whole population and had several limitations. First, the number of subjects in the included studies was still small, and only two studies used data from outside the Chinese population; thus, more large-scale studies are needed to assess the association between *ADIPOQ* +45T>G and MS. Second, environmental factors such as smoking, drinking, physical activity and family income might have a substantial influence on the progression of MS, but the lack of raw data makes it impossible for us to account for gene-environment interactions. Third, our analysis only focuses on *ADIPOQ* +*45*T>G; however, the development of MS is not due to a single gene, and there should be cross-talk between *ADIPOQ* +*45*T>G and other gene polymorphisms that should be considered^27^.

Given the complexity of the MS and the lack of clarity surrounding its definition, most reports to date have been restricted to the examination of the relationship between variant polymorphisms and the individual criteria of MS, rather than with the syndrome as a whole. For example, individuals carrying *ADIPOQ* +45T>G polymorphism were found to be associated with type 2 diabetes mellitus in Chinese, however, conflicting results have been reported in Asian and European subjects^57^,^58^. To our knowledge, this prospective study is the first to date to demonstrate the findings of studies about the association of *ADIPOQ* +45T>G with MS in the whole population together with a stratified analysis. There was only one prior meta-analysis, conducted by GAO M *et al*., that showed a significant relationship between *ADIPOQ* +45T>G and MS that differed from ours^9^. There might be several potential reasons for this difference. First, GAO M *et al*. performed their meta-analysis specifically in the Chinese population. In the present study, we focused on studies throughout the world that were up to date, and we presented a more comprehensive conclusion rather than studying a single ethnic group. Additionally, considering the distinct genetic backgrounds in the population, we further performed a subgroup analysis on East Asians. Second, GAO M *et al*.’s study only included 13 studies on *ADIPOQ* +45T>G in the Chinese population, whereas our study assessed 16 studies including Asian Indian and Thai populations. Furthermore, data from LI YP *et al*. were updated to the 2010 version. The larger sample size contributed to having greater statistical power to detect associations. Third, we also conducted a stratified analysis for different languages and diagnostic criteria as well as a subgroup analysis in East Asians to derive an accurate evaluation and validated our conclusions. Finally, and most importantly, we summarized the previous meta-analysis with ours and evaluated the pivotal role of *ADIPOQ* +45T>G in all potential metabolic-related disease risks including MS and metabolic-related disease. A clear risk of *ADIPOQ* +45T>G for obesity, cancer, NAFLD, T2DM, DN, hypertension, CAD, CHD and POVS was found. These disorders usually co-exist with each other^59^,^60^. This noteworthy finding led to the possible hypothesis that *ADIPOQ* +45T>G might be a common genetic factor behind these diseases and could explain the molecular mechanism of comorbidity in some patients.

Therefore, there exist some shared and distinct mechanisms that need to be clarified regarding MS, metabolic-related disease and their comorbidities. The answer might be somewhat as follows. First, the populations in the existing studies on MS and metabolic-related disease were so different that the confounding factors could not be controlled properly. Second, there must be a key individual characteristic on top of general factors (*ADIPOQ* +45T>G) that is present in MS and metabolic-related disease. According to the forest plot of our summarized analysis, although the *ADIPOQ* gene might be the “common soil” of individual MS components and other potential metabolic-related diseases, its contribution to MS as a whole appeared quite differently^61^. A genome wide association study (GWAS) suggested plasma adiponectin did not relate to genetic loci associated with MS parameters, indicating that MS is not modulated directly by the genetically determined adiponectin^29^,^62^. In fact, previous clinical observations categorized MS into two major components: one cluster was glucose/obesity/lipids, and the other was blood pressure^29^. These two groups significantly contributed to the genetic background of MS, indicating the heterogeneous nature of MS and the possibly distinct pathogenesis between MS with and without each cluster. In addition, metabolic-related diseases and MS might partially overlap with each other as specific clinical sub-phenotypes with a common genetic background. This could explain the inconsistent relationship found between *ADIPOQ* and individual MS parameters. Third, a well-designed follow-up cohort study including patients with both MS and metabolic-related disease that considers confounding factors and assesses a larger sample size with a focus on *ADIPOQ* +45T>G is required in the future.

In the secondary meta-analysis, various analytical methods demonstrated that there was no association between 5 μg/ml increments in adiponectin level and stroke risk (RR = 1.05, 95% CI = 1.00–1.10, *P* = 0.069). The conflicting results of the studies published previously may have existed for several reasons. First, the standards of measuring adiponectin level increments were not uniform in the included prospective and nested case-control cohort studies, which suggest it would be inappropriate to combine these measures in the overall RR. In our analysis, we stratified the analysis by the different measurement standards and evaluated the RR of stroke in 3 ways, as follows: stroke risk for the highest adiponectin level category compared with the lowest category; the overall RR per SD in log μg/ml of adiponectin; and the quantified RR of each 5 μg/ml increment in adiponectin level. Therefore, the conclusions we drew from these three analyses would be more accurate and reasonable. Second, serum adiponectin levels are related to various lipoproteins^63^. A limitation of the secondary analysis might be the limited data on HMW-adiponectin and the lack of data on other adiponectin fractions, which could have resulted in the influence of specific molecular-weight fractions of adiponectin on risk of stroke; this influence remains unclear.

In conclusion, our analysis suggested a weak positive association of *ADIPOQ* +45T>G with MS as well as a strong association with metabolic-related disease. As a result, the *ADIPOQ* gene could be treated as a promising physiological and pharmacological target in the prevention and treatment of disease with either a single component or comorbidity. Second, our analysis suggested that an elevated adiponectin level was not a causal factor contributing to an increase in the risk of stroke. Further large-scale studies in a rigorously defined cohort should be carried out to validate our findings.

## Additional Information

**How to cite this article**: Yuan, H.-P. *et al*. Association of Adiponectin Polymorphism with Metabolic Syndrome Risk and Adiponectin Level with Stroke Risk: A Meta-Analysis. *Sci. Rep.* **6**, 31945; doi: 10.1038/srep31945 (2016).

## Supplementary Material

Supplementary Information

## Figures and Tables

**Table 1:** Characteristics of Identified Studies on *ADIPOQ* +45T>G and MS risk.

Author	Year	Country/Ethnicity	Diagnostic Criteria	Genotyping Method	MS			Control			Language	QA	APN/(μg/ml)	
					TT	GT	GG	TT	GT	GG			MS	Control
YAO M^22^	2004	Chinese/EA	WHO,1999	PCR-RFLP	91	79	18	87	77	22	in Chinese	8	4.93 ± 3.31	9.29 ± 4.83
LIU DX^23^	2006	Chinese/EA	IDF, 2005	PCR-RFLP	73	66	17	69	53	11	in Chinese	7	NA	NA
LI YP^24^	2010	Chinese/EA	IDF, 2006	PCR-RFLP	69	54	14	56	60	15	in Chinese	6	NA	NA
ZHU XW^25^	2010	Chinese/EA	CDS, 2004	TaqMan	72	91	20	74	62	8	in Chinese	7	NA	NA
CAI Q^26^	2010	Chinese/EA	IDF, 2005	PCR-RFLP	15	18	5	26	20	4	in Chinese	6	NA	NA
Ranjith N^27^	2011	NA/SA	NCEP ATP III	TaqMan	208	81	6	134	50	6	in English	8	NA	NA
Ranjith N^27^	2011	NA/SA	IDF	TaqMan	204	79	7	138	52	5	in English	8	NA	NA
BU RF^28^	2011	Chinese/EA	CDS, 2004	TaqMan	76	97	22	79	67	10	in Chinese	7	6.85 ± 2.97	12.1 ± 5.75
Leu HB-1^29^	2011	Chinese/EA	ATP III	TaqMan	170	156	31	307	251	47	in English	8	NA	NA
Leu HB-2^29^	2011	Chinese/EA	ATP III	TaqMan	264	224	42	446	398	69	in English	8	NA	NA
HUANG FZ^30^	2012	Chinese/EA	IDF, 2005	PCR-RFLP	118	92	14	108	80	12	in Chinese	7	NA	NA
WANG SJ^21^	2012	Chinese/EA	WHO, 1999	PCR-RFLP	90	74	16	31	16	3	in Chinese	7	9.81 ± 0.55	13.32 ± 1.07
LI XT^31^	2012	Chinese/EA	CDS, 2004	PCR-RFLP	71	40	5	76	28	4	in English	7	NA	NA
CHEN F-1^32^	2012	Chinese/EA	CDS, 2004	PCR-RFLP	53	49	5	59	35	8	in Chinese	7	8.46 ± 5.96	10.87 ± 3.61
CHEN F-2^32^	2012	Chinese/EA	NA	PCR-RFLP	68	19	6	59	35	8	in Chinese	7	10.04 ± 6.39	NA
GAO M^9^	2013	Chinese/EA	IDF	PCR-RFLP	147	158	17	93	61	7	in English	8	NA	NA
SHEN J^* ^33	2013	Chinese/EA Chinese	CDS, 2004	PCR-RFLP	48	93	66	78	15	5	in Chinese	7	9.70 ± 1.46	11.85 ± 1.46
XU J^34^	2013	Chinese/EA	IDF, 2005	TaqMan	368	274	59	97	93	16	in Chinese	8	9.85 (9.35–10.38)	15.46 (14.13–16.90)
Suriyaprom K^35^	2014	Thailand/SEA	NCEP/ATP III	NA	84	NA	NA	102	NA	NA	in English	6	7.9 (7.0–9.2)	14.5 (12.7–16.2)

Note: MS = Metabolic Syndrome; APN = adiponectin; NA = Not Available; EA = East Asians; SA = South Asians; SEA = Southeast Asians; QA = Quality Assessment; ^*^the HUI nationality. Leu HB-1and Leu HB-2 represent two separate populations. CHEN F-1: metabolic syndrome; CHEN F-2: metabolic syndrome with coronary heart disease.

**Table 2:** Characteristics of the Identified Studies (N = 12) on Adiponectin and Risk of Stroke.

First author	Year	Cohort designation	Country	Follow-up (years)	Age, (Mean) (years)	% Men	Number of participants^a^	Number of cases	Cohort design
Söderberg S^36^	2004	MONICA	Sweden	4.9	25–74 (54.9)	57	828	276	Nested case–control
Matsumoto M^37^	2008	JMSCS	Japan	9.7	19–93 (66)	49	809	179	Nested case–control
David J Stott^38^	2009	PROSPER	UK	3.2	70–82 (75.9)	51	798	266	Nested case–control
Ogorodnikova AD^39^	2010	HaBPS	U.S.		50–59	0	1701	855	Nested case–control
P. Khalili^40^	2010	MPP	Sweden	27	(47)	100	3512	373	Prospective
Rajpathak SN^41^	2011	WHI-OS	U.S.	15–20	50–79 (68.7)	0	1944	972	Nested case–control
Prugger C^42^	2012	PRIME	Northern Ireland and France	10	50–59 (55.5)	100	285	95	Nested case–control
Wannamethee SG^43^	2013	BRHS	UK	9	60–79 (68.4)	100	3411	192	Prospective
Gardener H^44^	2013	NOMAS	U.S.	10	(69)	37	2900	269	Prospective
Bidulescu A^45^	2013	JHS	U.S.	6.2	21–94 (54 ± 13)	36	4571	87	Prospective
Men						100		31	
Women						0		56	
Kizer JR^46^	2013	CHS	U.S.	10.5	(74.4)	37	3290	492	Prospective
Arregui M^11^	2014	EPIC	Germany	8.2	(50.1)	37	2155	190	Nested case–control
Men							804	90	
Women							1351	80	

MONICA = Monitoring of Trends and Determinants in Cardiovascular Diseases; JMSCS = Jichi Medical School Cohort Study; PROSPER = Prospective Study of Pravastatin in the Elderly; HaBPS = Hormones and Biomarkers Predicting Stroke (HaBPS) ancillary study; MPP = Malmo Preventive Project; WHI-OS = Women’s Health Initiative Observational Study; PRIME = Prospective Epidemiological Study on Myocardial Infarction; BRHS = The British Regional Heart Study; NOMAS = the Northern Manhattan Study; JHS = Jackson Heart Study; CHS = Cardiovascular Health Study; EPIC = European Prospective Investigation into Cancer.

**Table 3:** Relative Risks of Stroke According to Serum Adiponectin Levels in the 12 Identified Studies.

First author	Year	Comparison	RR (95% CI)	Adjustment for Covariates
Söderberg S^36^	2004			Sex, age, date/type of health survey, region, BMI, smoking, hypertension, cholesterol, and diabetes mellitus
	Men	Q4 (≥17.3) vs. Q1 (<8.3)	1.08 (0.56–2.08)	
		Per 5 μg/ml	1.03 (0.89–1.21)	
	Women	Q4 (≥27.6) vs. Q1 (<14.5)	0.85 (0.44–1.63)	
		Per 5 μg/ml	0.97 (0.88–1.07)	
Matsumoto M^37^	2008	Q4 (>12.4; median, 14.35) *vs.* Q1 (<5.6; median, 4.25)	0.87 (0.49–1.54)	Age, sex, HDL cholesterol, triglyceride, BMI, current smoking, systolic blood pressure, and high-sensitivity CRP.
		Per standard-deviation increase in log μg/ml	1.06 (0.86–1.31)	
		Per 5 μg/ml	0.95 (0.69–1.31)	
David J Stott^38^	2009			
		Per standard-deviation increase (4.89 μg/ml)	0.86 (0.72–1.03)	
		Per standard-deviation increase (4.89 μg/ml)	0.78 (0.62–0.97)*	
Ogorodnikova AD^39^	2010	Q4 (>18.8; median, 22.65) *vs.* Q1 (<5.2; median, 2.3)	1.25 (0.88–1.79)	Age, race/ethnicity, BMI groups, type 2 diabetes, smoking, hypertension, LDL-C, HDL-C, METs, CRP, and aspirin use.
		Per 5 μg/ml	1.08 (0.99–1.17)	
P. Khalili^40^	2010	Q5 (median, 16.57) *vs.* Q1 (median, 2.37)	0.98 (0.65–1.47)*	
		Per 5 μg/ml	1.04 (0.95–1.13)*	
Rajpathak SN^41^	2011	Q4 (median, 46) *vs.* Q1 (median, 14.8)	1.16 (0.82–1.63)*	Age, ethnicity, BMI, current smoking, physical activity, NSAIDs use, hypertension medication use, systolic blood pressure, history of coronary and artery diseases, HDL cholesterol, triglyceride, diabetes, and waist circumference.
		Per 5 μg/ml	1.01 (0.97–1.06)*	
Prugger C^42^	2012			Systolic blood pressure, antihypertensive treatment, cigarette smoking, alcohol consumption, total cholesterol, high-density lipoprotein cholesterol, waist circumference, diabetes mellitus, and high-sensitivity C-reactive protein.
		Per standard-deviation increase	1.53 (1.01–2.34)*	
		Per 5 μg/ml	1.40 (1.14–1.71)*	
Wannamethee SG^43^	2013	Q4 (>10.839, median, 12.775) *vs.* Q1 (<4.346, median, 3.037)	0.73 (0.48–1.10)	Age, BMI, diabetes mellitus, angina, atrial fibrillation, smoking, social class, alcohol intake, physical activity, lung function, systolic blood pressure, and use of antihypertensive drugs.
		Per 5 μg/ml	0.94 (0.75–1.19)	
Gardener H^44^	2013	Q4 (13.8–53.3, median, 33.55) *vs.* Q1 (2.1–7.0, median, 4.55)	1.64 (1.01–2.63)	Age, sex, race/ethnicity, smoking, hypertension, diabetes, low density lipoprotein cholesterol, high density lipoprotein cholesterol, triglycerides, waist circumference, moderate alcohol use, moderate-heavy physical activity, previous cardiac disease history, and hs-CRP.
		Per 5 μg/ml	1.16 (1.03–1.31)	
Bidulescu A^45^	2013			Age, body mass index, systolic blood pressure, blood pressure medication, HDL-cholesterol, triglycerides, C-reactive protein, insulin resistance by HOMA-IR, smoking, and physical activity.
	Men	Per standard-deviation increase in log μg/ml	1.18 (0.79–1.74)*	
	Women	Per standard-deviation increase in log μg/ml	1.41 (1.04–1.91)*	
Kizer JR^46^	2013	Per standard-deviation increase (7.9 μg/ml)	<20 μg/ml, 1.10 (0.89–1.35)*	Age, sex, race, BMI, income, education, centre, smoking status, alcohol use, systolic blood pressure, antihypertensive medication, oestrogen replacement therapy, eGFR, aspirin use, health status, albumin, subclinical CVD, pre/diabetes mellitus, LDL-cholesterol, HDL cholesterol, triglycerides, and hs-CRP.
		Per standard-deviation increase (7.9 μg/ml)	≥20 μg/ml, 1.16 (0.97–1.38)*	
		Per 5 μg/ml	1.07 (0.98–1.16)*	
Arregui M^11^	2014	Q3 (median, 9.4) *vs.* Q1 (median, 3.5)	1.94 (1.22–3.06)*	Age, waist circumference, smoking status (never smoker, former smoker, current smoker <20 cigarettes per day, current smoker ≥20 cigarettes per day), sports activity (<2 h/week, ≥2 h/week), education (vocational school or less, technical school, university), alcohol consumption (men: 0, >0–12, >12–24, >24; women: 0, >0–6, >6–12, >12 g/day), prevalent hypertension, fasting status (yes/no), prevalent diabetes, HDL-cholesterol, triglycerides, and hs-CRP.
	Men	Per 5 μg/ml	1.15 (0.76–1.73)*	
	Women	Per 5 μg/ml	1.30 (1.02–1.67)*	

^*^Estimates specific to ischemic stroke; Q = quintiles or quartiles.

## References

[b1] Kaur J. A comprehensive review on metabolic syndrome. Cardiol Res Pract. 943162 (2014). 24711954 10.1155/2014/943162 PMC3966331

[b2] Taylor J. Y. . An overview of the genomics of metabolic syndrome. J Nurs Scholarsh 45(1), 52–59 (2013). 23368731 10.1111/j.1547-5069.2012.01484.x PMC3594572

[b3] Gu H. F. . Single nucleotide polymorphisms in the proximal promoter region of the adiponectin (APM1) gene are associated with type 2 diabetes in Swedish caucasians. Diabetes 53 Suppl 1, S31–S35 (2004). 14749263 10.2337/diabetes.53.2007.s31

[b4] Stumvoll M. . Association of the T-G polymorphism in adiponectin (exon 2) with obesity and insulin sensitivity: interaction with family history of type 2 diabetes. Diabetes 51(1), 37–41 (2002). 11756320 10.2337/diabetes.51.1.37

[b5] Henneman P. . Genetic architecture of plasma adiponectin overlaps with the genetics of metabolic syndrome-related traits. Diabetes Care 33(4), 908–913 (2010). 20067957 10.2337/dc09-1385 PMC2845050

[b6] Ruan H. & Dong L. Q. Adiponectin signaling and function in insulin target tissues. J Mol Cell Biol. [Epub ahead of print] (2016). 10.1093/jmcb/mjw014 PMC4816150 26993044

[b7] Chu H. . AdipoQ polymorphisms are associated with type 2 diabetes mellitus: a meta-analysis study. Diabetes Metab Res Rev 29(7), 532–545 (2013). 23653335 10.1002/dmrr.2424

[b8] Melistas L. . Association of the +45T>G and +276G > T polymorphisms in the adiponectin gene with insulin resistance in nondiabetic Greek women. Eur J Endocrinol 161(6), 845–852 (2009). 19755407 10.1530/EJE-09-0492 PMC2896503

[b9] Gao M. . Association of genetic variants in the adiponectin gene with metabolic syndrome: a case-control study and a systematic meta-analysis in the Chinese population. PLoS One 8(4), e58412 (2013). 23593121 10.1371/journal.pone.0058412 PMC3617191

[b10] Kanhai D. A. . Adiponectin and incident coronary heart disease and stroke. A systematic review and meta-analysis of prospective studies. Obes Rev 14(7), 555–567 (2013). 23495931 10.1111/obr.12027

[b11] Arregui M. . Adiponectin and risk of stroke: prospective study and meta-analysis. Stroke 45(1), 10–17 (2014). 24203850 10.1161/STROKEAHA.113.001851

[b12] Hao G. . Serum total adiponectin level and the risk of cardiovascular disease in general population: a meta-analysis of 17 prospective studies. Atherosclerosis 228(1), 29–35 (2013). 23489345 10.1016/j.atherosclerosis.2013.02.018

[b13] Moher D., Liberati A., Tetzlaff J. & Altman D. G. Preferred reporting items for systematic reviews and meta-analyses: the PRISMA statement. PLoS Med 6(7), e1000097 (2009). 19621072 10.1371/journal.pmed.1000097 PMC2707599

[b14] Liberati A. . The PRISMA statement for reporting systematic reviews and meta-analyses of studies that evaluate health care interventions: explanation and elaboration. PLoS Med 6(7), e1000100 (2009). 19621070 10.1371/journal.pmed.1000100 PMC2707010

[b15] Wells G. A., D O’Connell B. S., Peterson J., Welch V., Losos M. & Tugwell P. The Newcastle-Ottawa Scale (NOS) for assessing the quality of nonrandomised studies in meta-analyses. Available at: www.ohri.ca/programs/clinical_epidemiology/oxford.asp. (Accessed: 1st May 2016) (2011).

[b16] Li K. . Association of two polymorphisms rs2910164 in miRNA-146a and rs3746444 in miRNA-499 with rheumatoid arthritis: a meta-analysis. Hum Immunol 75(7), 602–608 (2014). 24824381 10.1016/j.humimm.2014.05.002

[b17] Qiu M. T. . Hsa-miR-499 rs3746444 polymorphism contributes to cancer risk: a meta-analysis of 12 studies. PLoS One 7(12), e50887 (2012). 23236400 10.1371/journal.pone.0050887 PMC3517592

[b18] Yuan H. . Serum Uric Acid Levels and Risk of Metabolic Syndrome: A Dose-Response Meta-Analysis of Prospective Studies. J Clin Endocrinol Metab 100(11), 4198–4207 (2015). 26308292 10.1210/jc.2015-2527

[b19] Higgins J. P. & Thompson S. G. Quantifying heterogeneity in a meta-analysis. Stat Med 21(11), 1539–1558 (2002). 12111919 10.1002/sim.1186

[b20] Higgins JPT, G.S.e., Cochrane Handbook for Systematic Reviews of Interventions Version 5.1.0 [updated March 2011]. The Cochrane Collaboration, Available at: www.cochrane-handbook.org. (Accessed: 1st May 2016) (2011).

[b21] Wang S. J., Jia W. P., Bao Y. Q. & JQ L. Association of adiponectin gene polymorphism with metabolic syndrome. J Chinese Practical Diagnosis and Therapy 26(7), 659–661 (2012).

[b22] Yao M. Diploma Thesis, Chinese Academy of Medical Science & Peking Union Medical College, 2004.

[b23] Liu D. X., Hua Q. i., Guo J. C. & Liu R. K. Difference of adiponectin gene exon 2 Glyl5Gly genotype and allele frequency between patients with metabolic syndrome and normal persons. Chin J Clin Rehabilitation 10(6), 4–6 (2006).

[b24] Li Y. P. . Adiponectin gene SNP11377 and SNP-4522 haplotypes are related to metabolic syndrome. Basic Clin Med 30(12), 1288–1292 (2010).

[b25] Zhu X. W. . Studies on adiponectin gene polymorphisms in patients with metabolic syndrome. Shandong Med J 50(29), 10–12 (2010).

[b26] Cai Q., Liu H. J., Chen T. & MC Z. Mononucleotide polymorphism at +45 and +276 site of adiponectin gene in aircrew with metabolic syndrome. Clin J Med Officers 38(5), 813–815 (2010).

[b27] Ranjith N., Pegoraro R. J. & Shanmugam R. Obesity-associated genetic variants in young Asian Indians with the metabolic syndrome and myocardial infarction. Cardiovasc J Afr 22(1), 25–30 (2011). 21298202 10.5830/CVJA-2010-036 PMC3736384

[b28] Bu R. F. . The association of adiponectin gene polymorphism and serum adiponectin level with metabolic syndrome in older. Chin J Gerontology 31, 4112–4114 (2011).

[b29] Leu H. B. . Adiponectin gene polymorphism is selectively associated with the concomitant presence of metabolic syndrome and essential hypertension. PLoS One 6(5), e19999 (2011). 21637762 10.1371/journal.pone.0019999 PMC3103519

[b30] Huang F. Z., Liu J., Pang D. & PL B. The association of adiponectin gene SNP +276 and SNP +45 with metabolic syndrome. Chinese J of Endocrinology and Metabolism 28(11), 911–913 (2012).

[b31] Li X. T. . Association of the adiponectin gene (ADIPOQ) +45 T. G polymorphism with the metabolic syndrome among Han Chinese in Sichuan province of China. Asia Pac J Clin Nutr 21(2), 296–301 (2012). 22507618

[b32] Chen F. . Study on the correlation of adiponectin gene polymorphism and coronary heart disease in the patients with metabolic syndrome in Han nationlity population in Ningxia. Clin J Crit Care Med 32(1), 24–29 (2012).

[b33] Shen J. . Relation of metabolic syndrome with plasma adiponectin level and gene polymorphism in Ningxia Hui subjects. Chin J Geriatr Heart Brain Vessel Dis 15(1), 7–10 (2013).

[b34] Xu J. . Association of adiponectin gene polymorphrism with metabolic syndrome in older Han adults from major cities in China. Journal of Hygiene Research 42(3), 353–359 (2013). 23805506

[b35] Suriyaprom K., Phonrat B. & Tungtrongchitr R. Association of adiponectin gene -11377C > G polymorphism with adiponectin levels and the metabolic syndrome in Thais. Asia Pac J Clin Nutr 23(1), 167–173 (2014). 24561985 10.6133/apjcn.2014.23.1.01

[b36] Soderberg S. . Leptin, but not adiponectin, predicts stroke in males. J Intern Med 256(2), 128–136 (2004). 15257725 10.1111/j.1365-2796.2004.01351.x

[b37] Matsumoto M., Ishikawa S. & Kajii E. Association of adiponectin with cerebrovascular disease: a nested case-control study. Stroke 39(2), 323–328 (2008). 18096839 10.1161/STROKEAHA.107.497552

[b38] Stott D. J. . Adipocytokines and risk of stroke in older people: a nested case-control study. Int J Epidemiol 38(1), 253–261 (2009). 18952622 10.1093/ije/dyn215

[b39] Ogorodnikova A. D. . High-molecular-weight adiponectin and incident ischemic stroke in postmenopausal women: a Women’s Health Initiative Study. Stroke 41(7), 1376–1381 (2010). 20508194 10.1161/STROKEAHA.109.576546 PMC2907159

[b40] Khalili P. . Total adiponectin does not predict cardiovascular events in middle-aged men in a prospective, long-term follow-up study. Diabetes Metab 36(2), 137–143 (2010). 20153676 10.1016/j.diabet.2009.10.004

[b41] Rajpathak S. N. . Resistin, but not adiponectin and leptin, is associated with the risk of ischemic stroke among postmenopausal women: results from the Women’s Health Initiative. Stroke 42(7), 1813–1820 (2011). 21546486 10.1161/STROKEAHA.110.607853 PMC4059356

[b42] Prugger C. . Adipocytokines and the risk of ischemic stroke: the PRIME Study. Ann Neurol 71(4), 478–486 (2012). 22522440 10.1002/ana.22669

[b43] Wannamethee S. G. . Adiposity, adipokines, and risk of incident stroke in older men. Stroke 44(1), 3–8 (2013). 23192755 10.1161/STROKEAHA.112.670331

[b44] Gardener H. . Adiponectin and risk of vascular events in the Northern Manhattan study. Atherosclerosis 226(2), 483–489 (2013). 23245751 10.1016/j.atherosclerosis.2012.11.020 PMC3546281

[b45] Bidulescu A. . Associations of adiponectin and leptin with incident coronary heart disease and ischemic stroke in african americans: the jackson heart study. Front Public Health 1, 16 (2013). 24350185 10.3389/fpubh.2013.00016 PMC3854845

[b46] Kizer J. R. . Total and high-molecular-weight adiponectin and risk of coronary heart disease and ischemic stroke in older adults. J Clin Endocrinol Metab 98(1), 255–263 (2013). 23162097 10.1210/jc.2012-2103 PMC3537098

[b47] Scherer P. E. Adipose tissue: from lipid storage compartment to endocrine organ. Diabetes 55(6), 1537–1545 (2006). 16731815 10.2337/db06-0263

[b48] Santaniemi M., Kesaniemi Y. A. & Ukkola O. Low plasma adiponectin concentration is an indicator of the metabolic syndrome. Eur J Endocrinol 155(5), 745–750 (2006). 17062891 10.1530/eje.1.02287

[b49] Vasseur F. . Single-nucleotide polymorphism haplotypes in the both proximal promoter and exon 3 of the APM1 gene modulate adipocyte-secreted adiponectin hormone levels and contribute to the genetic risk for type 2 diabetes in French Caucasians. Hum Mol Genet 11(21), 2607–2614 (2002). 12354786 10.1093/hmg/11.21.2607

[b50] Prakash J., Mittal B., Awasthi S. & Srivastava N., Association of adiponectin gene polymorphism with adiponectin levels and risk for insulin resistance syndrome. Int J Prev Med 6, 31 (2015). 25949781 10.4103/2008-7802.154773 PMC4410438

[b51] Guzman-Ornelas M. O. . Association of ADIPOQ +45T >G polymorphism with body fat mass and blood levels of soluble adiponectin and inflammation markers in a Mexican-Mestizo population. Diabetes Metab Syndr Obes 5, 369–378 (2012). 23118546 10.2147/DMSO.S35434 PMC3484511

[b52] Wu J., Liu Z., Meng K. & Zhang L. Association of adiponectin gene (ADIPOQ) rs2241766 polymorphism with obesity in adults: a meta-analysis. PLoS One 9(4), e95270 (2014). 24740426 10.1371/journal.pone.0095270 PMC3989273

[b53] BC Z., WJ L., WL C. & YW X. Serum total adiponectin level and risk of cardiovascular disease in Han Chinese populations: a meta-analysis of 17 case-control studies. Clin Endocrinol (Oxf) 77(3), 370–378 (2012). 21995850 10.1111/j.1365-2265.2011.04260.x

[b54] Thompson S. G. Why sources of heterogeneity in meta-analysis should be investigated. BMJ 309(6965), 1351–1355 (1994). 7866085 10.1136/bmj.309.6965.1351 PMC2541868

[b55] Munafo M. R. & Flint J. Meta-analysis of genetic association studies. Trends Genet 20(9), 439–444 (2004). 15313553 10.1016/j.tig.2004.06.014

[b56] Fan Y. . Association between ADIPOQ +45T>G polymorphism and type 2 diabetes: a systematic review and meta-analysis. Int J Mol Sci 16(1), 704–723 (2015). 25561226 10.3390/ijms16010704 PMC4307270

[b57] Vimaleswaran K. S. . A novel association of a polymorphism in the first intron of adiponectin gene with type 2 diabetes, obesity and hypoadiponectinemia in Asian Indians. Hum Genet 123(6), 599–605 (2008). 18465144 10.1007/s00439-008-0506-8

[b58] Fan Y. . Association between ADIPOQ +45T>G polymorphism and type 2 diabetes: a systematic review and meta-analysis. Int J Mol Sci 16(1), 704–723 (2014). 25561226 10.3390/ijms16010704 PMC4307270

[b59] Valderas J. M. . Defining comorbidity: implications for understanding health and health services. Ann Fam Med 7(4), 357–363 (2009). 19597174 10.1370/afm.983 PMC2713155

[b60] Jakovljevic M. & Ostojic L. Comorbidity and multimorbidity in medicine today: challenges and opportunities for bringing separated branches of medicine closer to each other. Psychiatr Danub 25 Suppl 1, 18–28 (2013). 23806971

[b61] Stern M. P. Diabetes and cardiovascular disease. The “common soil” hypothesis. Diabetes 44(4), 369–374 (1995). 7698502 10.2337/diab.44.4.369

[b62] Heid I. M. . Clear detection of ADIPOQ locus as the major gene for plasma adiponectin: results of genome-wide association analyses including 4659 European individuals. Atherosclerosis 208(2), 412–420 (2010). 20018283 10.1016/j.atherosclerosis.2009.11.035 PMC2845297

[b63] Cnop M. . Relationship of adiponectin to body fat distribution, insulin sensitivity and plasma lipoproteins: evidence for independent roles of age and sex. Diabetologia 46(4), 459–469 (2003). 12687327 10.1007/s00125-003-1074-z

