# Supplementary material for: Association of Adiponectin Polymorphism with Metabolic Syndrome Risk and Adiponectin Level with Stroke Risk: A Meta-Analysis
Source: Sci Rep. 2016 Aug 31;6:31945. doi: 10.1038/srep31945 (PMC5005996; doi:10.1038/srep31945)
Supplement: Supplementary Information [file srep31945-s1.pdf]

Association of Adiponectin Polymorphism with Metabolic Syndrome Risk and Adiponectin Level with Stroke Risk: A Meta-Analysis

Hui-Ping Yuan[1§], Liang Sun[1], Xing-Hui Li[1], Fu-Gang Che[1], Xiao-Quan Zhu[1], Fan Yang[1], Jing Han[1], Chun-Yuan Jia[1], Ze Yang[1§]

[1]The Key Laboratory of Geriatrics, Beijing Hospital, National Center of Gerontology & Beijing Institute of Geriatrics, Ministry of Health, Beijing 100730, P.R. China.

Supplementary Data

Supplementary Table S1. Stratified Meta-analyses of Adiponectin Levels per 5 μg/ml Increment and Risk of Stroke.

Supplementary Table S2. Characteristics of the Identified Studies of Meta-Analyses on ADIPOQ +45T>G and Risk of Metabolic-related Disease.

Supplementary Table S3. Scale for Methodological Quality Assessment for Identified Studies on ADIPOQ +45T>G and MS risk.

Supplementary Table S4. Quality Assessment of Included Studies on Adiponectin and Risk of Stroke.

Supplementary Fig. S1. Flow chart of literature selection for the meta-analysis.

Supplementary Fig. S2. Risk of incident stroke for the highest compared with the lowest category of serum adiponectin level.

Supplementary Fig. S3. Stratified meta-analysis for *ADIPOQ* +45T>G and the risk of metabolic syndrome.

Supplementary Fig. S4. Publication bias and sensitivity analysis for identified studies of two meta-analyses.

Supporting References

Supplementary Table S1. Stratified Meta-Analyses of Adiponectin Levels per 5 μg/ml Increment and Risk of Stroke[a].

Characteristic	Data Points, No.	Summary RR (95%CI)[b]	*P* Value for Heterogeneity	I^2 %	*P* Value for meta-regression[c]
All studies	13[a]	1.05 (1.00-1.10)	0.069	54.6	
Study design					0.952
Nested case-control	9	1.05 (0.97-1.13)	0.01	60.0	
Prospective	4	1.06 (0.99-1.14)	0.16	42.1	
Indicators of participant characteristics					
Country					0.851
Europe	8	1.04 (0.95-1.15)	0.006	64.7	
US	4	1.06 (1.01-1.12)	0.11	50.4	
Japan	1	0.95 (0.69-1.31)	NA	NA	
Mean age					0.264
<60	7	1.09 (1.00-1.18)	0.033	56.2	
⩾60	6	1.02 (0.95-1.09)	0.048	55.3	
Sex					0.699
Men	5	1.08 (0.94-1.22)	0.033	61.9	
Women	4	1.04 (0.97-1.11)	0.082	55.3	
Whole	4	1.03 (0.91-1.16)	0.048	62	
Follow-up duration (years)					0.144
<10	7	0.99 (0.90-1.08)	0.173	33.4	
⩾10	6	1.08 (1.02-1.15)	0.016	64.3	
Measure of association					0.125
Risk ratio	3	1.02 (0.95-1.09)	0.158	45.8	
Odds ratio	3	0.96 (0.89-1.05)	0.318	12.7	
Hazard ratio	7	1.13 (1.04-1.22)	0.094	44.5	

[a]Analysis based on 11 studies and 13 data points, because men and women were included separately for the study reported. [b]Pooled RRs of MS for each 5 μg/ml increase in adiponectin within the strata of each study characteristic are indicated. [c]Represents the test for significance of the effect across strata.

Supplementary Table S2. Characteristics of the Identified Studies of Meta-Analyses on ADIPOQ +45T>G and Risk of Metabolic-related Disease.

Author	Year	Components	Population	OR(95%CI)	*p* [a]	Model [b]	Effects Model [c]	Cases	Controls	No. of Studies
WU JJ[1]	2014	obesity	Mixed	1.39(1.11-1.73)	0.004	additive	Fixed	1888	2047	18
WU JJ[1]	2014	obesity	Chinese	1.54(1.19-2.00)	0.001	additive	Fixed	830	1010	11
YANG Y[2]	2013	cancer[d]	Mixed	0.768(0.63-0.94)	0.011	recessive	Fixed	5656	6321	9
YE CC[3]	2013	CRC	Asians, Caucasians	1.22(1.05-1.43)	0.010	heterozygote	Fixed	1250	1525	5
WANG J[4]	2014	NAFLD	Asians, Caucasians	1.48(1.07-2.06)	0.020	additive	Fixed	644	895	7
CHEN J[5]	2012	T2DM	Chinese	1.41(1.20-1.67)	<0.001	additive	Random	3737	3247	19
LIN Z[6]	2014	DN	Caucasians	1.12(1.01-1.25)	0.038	allele contrast	Fixed	2654	7710	9
XI B[7]	2012	hypertension	Chinese	1.22(1.01-1.48)	NA	recessive	Fixed	1812	2631	6
ZHOU DH[8]	2014	CVD	Caucasians	1.22(1.08-1.39)	0.002	allele contrast	Random	12378	19368	28
ZHANG	2012	CVD	Mixed	1.22(1.07-1.39)	0.004	additive	Random	6398	10829	24

H[BC, 2012 #369]										
ZHANG H[BC, 2012 #369]	2012	CHD	Mixed	1.29(1.09-1.52)	0.004	additive	Random	4685	5881	17
GAO LL[10]	2012	POVS	Mixed	1.36(1.12-1.65)	0.002	allele contrast	Fixed	792	1322	8
YUAN HP	2014	MS	Mixed	1.30(1.03-1.65)	0.027	dominant	Fixed	4113	3637	16

Note: MS: Metabolic syndrome; CRC: colorectal cancer; NAFLD: non alcoholic fatty liver disease; T2DM: type 2 diabetes; DN: diabetic nephropathy; CVD: cardiovascular disease; CHD: coronary heart disease; POVS: polycystic ovary syndrome; NA = not available;

[a] *P* value calculated based on the corresponding models; [b] the most significant genetic model; [c] effects model for quantitatively synthesis of individual study; [b] prostate cancer, colorectal cancer, lung cancer, breast cancer and pancreas cancer are included.

Supplementary Table S3. Scale for Methodological Quality Assessment for Identified Studies on ADIPOQ +45T>G and MS risk.

**Criteria**	**Score**
1.Representativeness of cases	
MS diagnosed criteria to acknowledged criteria	2
Mentioned the diagnosed criteria but not specifically described	1
Not described	0
2.Source of controls	
Population or community based	3
Hospital-based MS-free controls	2
Healthy volunteers without total description	1
MS-free controls with related diseases	0.5
Not described	0
3.Sample size	
>400	2
200-400	1
<200	0
4.Quality control of genotyping methods	
Repetition of partial/total tested samples with a different method	2
Repetition of partial/total tested samples with the same method	1
Not described	0
5.Hardy-Weinberg equilibrium (HWE)	
Hardy-Weinberg equilibrium in control subjects	1
Hardy-Weinberg disequilibrium in control subjects	0

Supplementary Table S4. Quality Assessment of Included Studies on Adiponectin and Risk of Stroke.

Study	Selection	Comparability	Outcome/Exposure	Overall quality
Söderberg S[11]	4	2	3	9
Matsumoto M[12]	4	2	2	8
David J Stott[13]	3	2	2	7
Ogorodnikova AD[14]	3	2	3	8
P. Khalili[15]	3	1	2	6
Rajpathak SN[16]	3	2	3	8
Prugger C[17]	3	1	3	7
Wannamethee SG[18]	4	1	3	8
Gardener H[19]	4	1	3	8
Bidulescu A[20]	3	1	2	6
Kizer JR[21]	3	2	2	7
Arregui M[22]	3	1	2	6

Supplementary Fig. S1. Flow chart of literature selection for the meta-analysis.

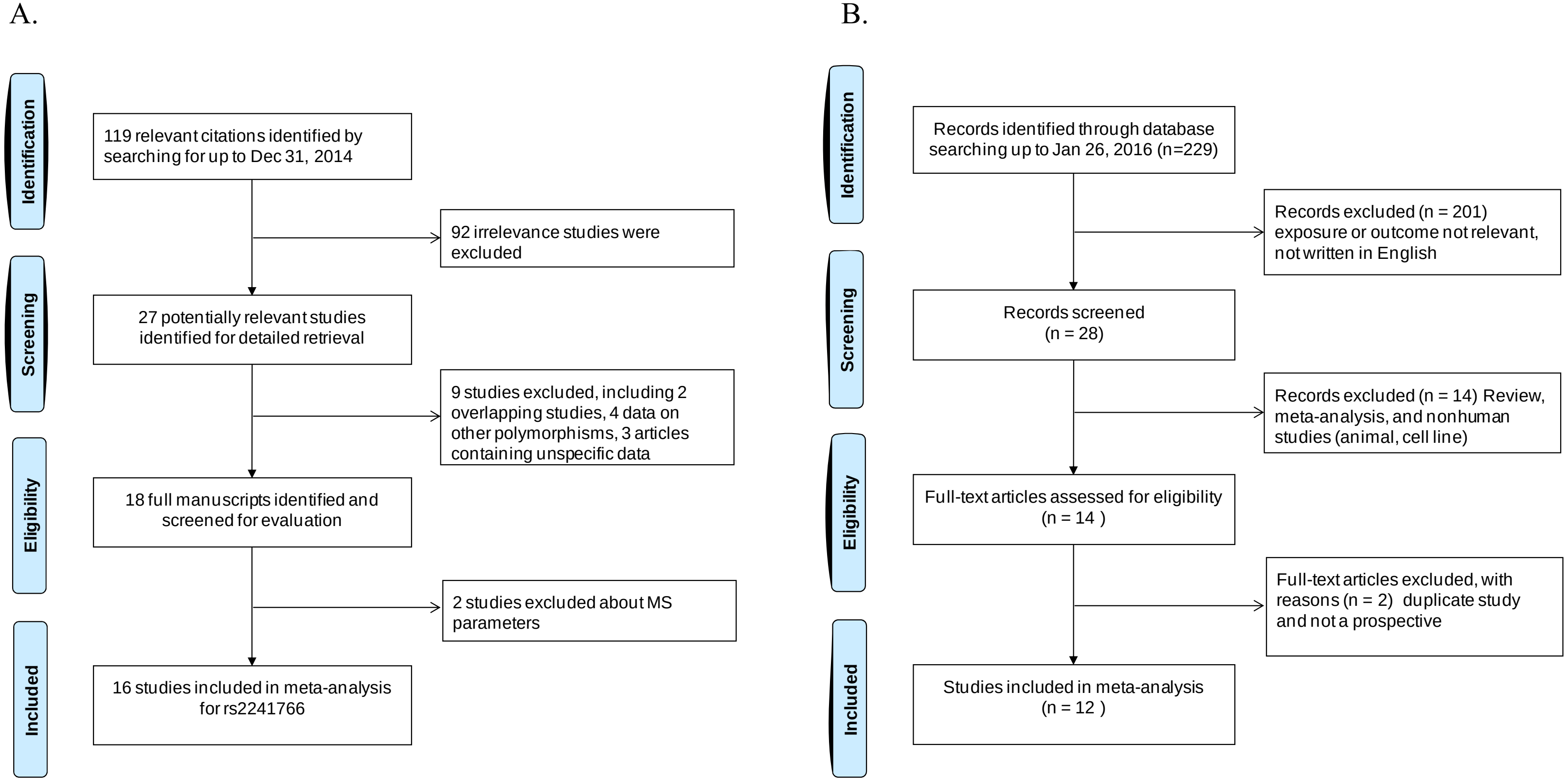

Flow diagram of eligible studies selection: articles were identified up to Dec 31, 2014 and Jan 26, 2016 for *ADIPOQ* +45T>G & MS (A) and adiponectin & stroke (B), respectively.

Supplementary Fig. S2. Risk of incident stroke for the highest compared with the lowest category of serum adiponectin level.

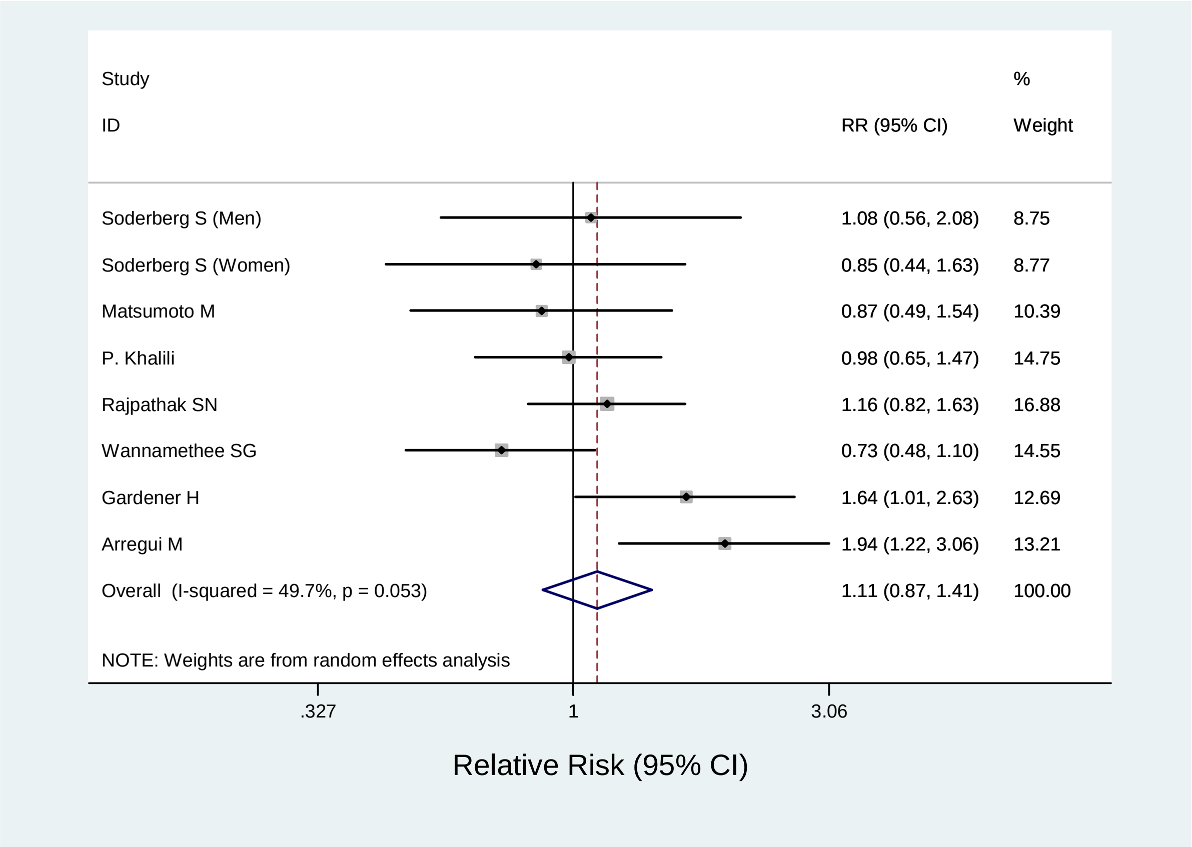

Random-effects model analysis for overall RR (1.11, 95% CI=0.87-1.41) of incident stroke for the highest compared with the lowest category of adiponectin level. The square sizes are proportional to the weight of each study in the meta-analysis; the horizontal lines represent 95% CIs; the diamond represents the overall RR with its 95% CI.

Supplementary Fig. S3. Stratified meta-analysis for *ADIPOQ* +45T>G and the risk of metabolic syndrome.

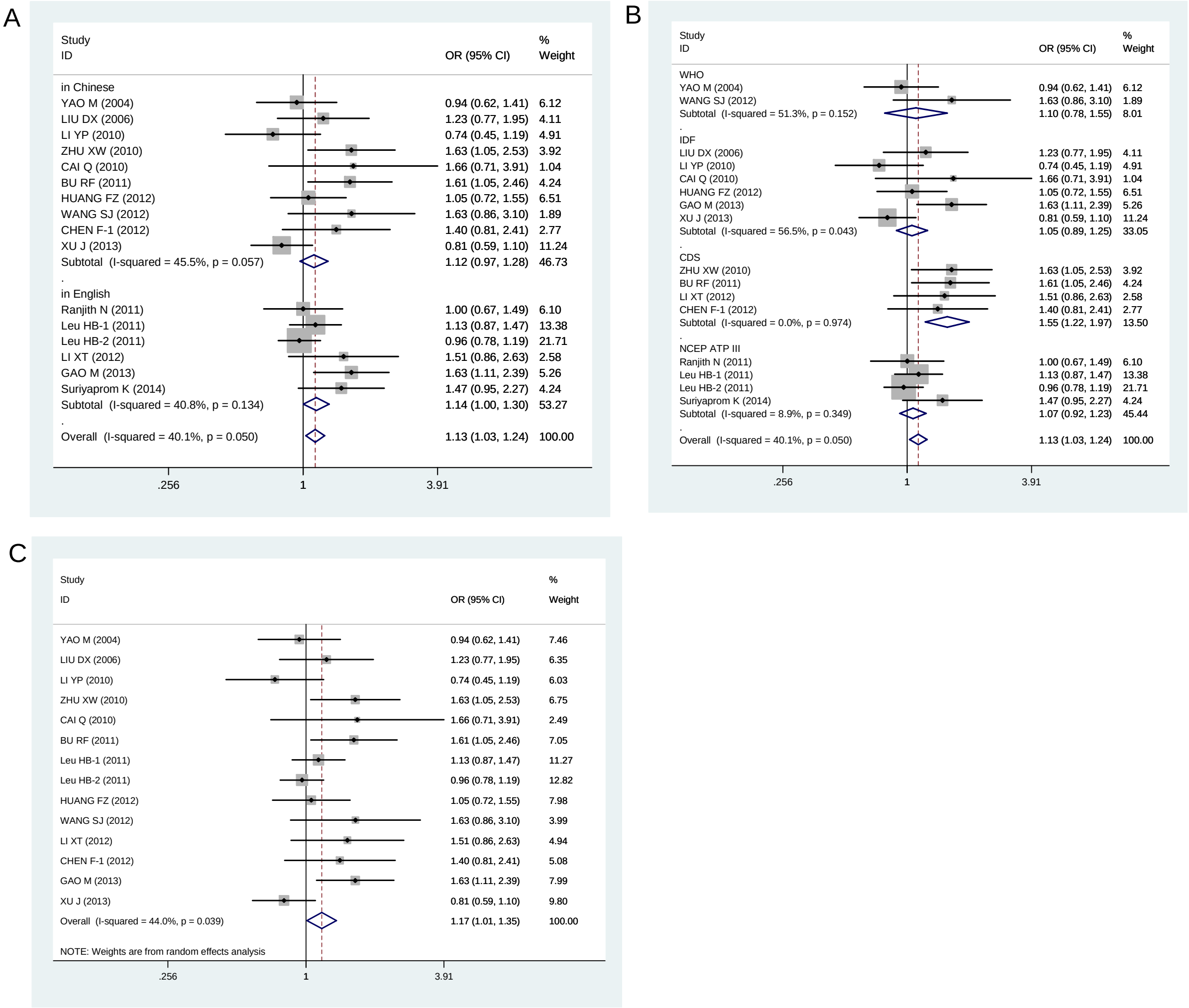

Stratified analyses for *ADIPOQ* +45T>G and the risk of MS. The square sizes are proportional to the weight of each study in the meta-analysis; the horizontal lines represent 95% CIs; the diamond represents the overall RR with its 95% CI.

Supplementary Fig. S4. Publication bias and sensitivity analysis for identified studies of two meta-analyses.

A.

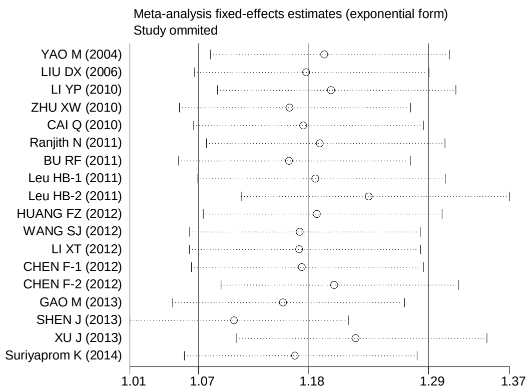

B.

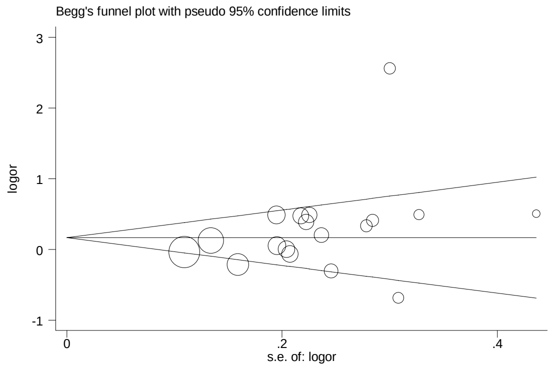

C.

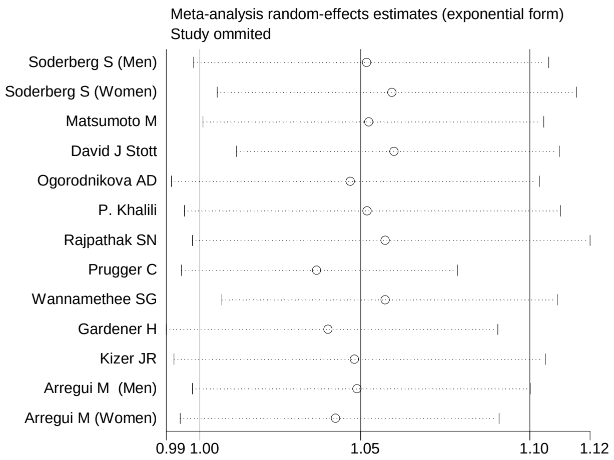

D.

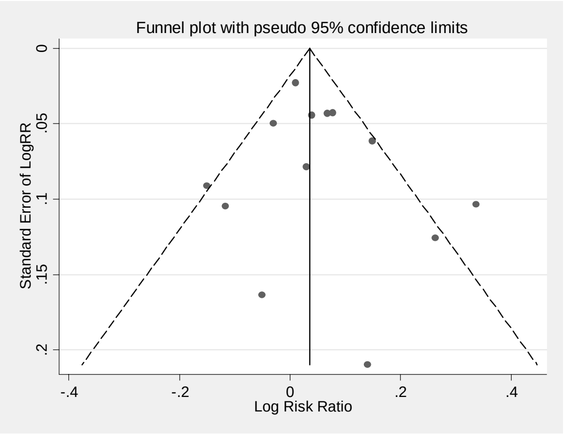

(A) Sensitivity analysis of the primary analysis (*ADIPOQ* +45T>G & MS): Horizontal line means effect size. Abbreviations: s.e: standardized effect; (B) Bgger's Funnel plot of the primary analysis to assess publication bias (*ADIPOQ* +45T>G & MS): Each point represents a separate study included in this meta-analysis. s.e: standardized effect. Leu HB-1and Leu HB-2 represents two separate populations. CHEN F-1: MS; CHEN F-2: MS with CHD; (C) Sensitivity analysis of the secondary analysis (adiponectin & stroke): Horizontal line means effect size. Abbreviations: s.e: standardized effect; (D) Funnel plot of the secondary analysis (adiponectin & stroke) to assess publication bias: Each point represents a separate study included in this meta-analysis. s.e: standardized effect.

Supporting References

1. Wu, J., Liu, Z., Meng, K., & Zhang, L., Association of adiponectin gene (ADIPOQ) rs2241766 polymorphism with obesity in adults: a meta-analysis. *PLoS One* **9** (4), e95270 (2014).
2. Yang, Y. *et al.*, ADIPOQ gene polymorphisms and cancer risk: a meta-analysis. *Cytokine* **61** (2), 565-571 (2013).
3. Ye, C. *et al.*, Meta-analysis of adiponectin polymorphisms and colorectal cancer risk. *Int J Med Sci* **10** (9), 1113-1120 (2013).
4. Wang, J. *et al.*, Adiponectin polymorphisms and non-alcoholic fatty liver disease risk: A meta-analysis. *J Gastroenterol Hepatol* **29** (7), 1396-1405 (2014).
5. Jing, C., Xueyao, H., & Linong, J., Meta-analysis of association studies between five candidate genes and type 2 diabetes in Chinese Han population. *Endocrine* **42** (2), 307-320 (2012).
6. Lin, Z., Huang, G., Zhang, J., & Lin, X., Adiponectin gene polymorphisms and susceptibility to diabetic nephropathy: a meta-analysis. *Ren Fail* **36** (3), 478-487 (2014).
7. Xi, B. *et al.*, Common polymorphisms (rs2241766 and rs1501299) in the ADIPOQ gene are not associated with hypertension susceptibility among the Chinese. *Mol Biol Rep* **39** (9), 8771-8775 (2012).
8. Zhou, D. *et al.*, Association between the adiponectin +45T>G genotype and risk of cardiovascular disease: a meta-analysis. *Heart Lung Circ* **23** (2), 159-165 (2014).
9. Zhang, H., Mo, X., Hao, Y., & Gu, D., Association between polymorphisms in the adiponectin gene and cardiovascular disease: a meta-analysis. *BMC Med Genet* **13**, 40 (2012).
10. Gao, L. *et al.*, Association of the T45G and G276T polymorphisms in the adiponectin gene with PCOS: A meta-analysis. *Gynecol Endocrinol* **28** (2), 106-110 (2012).
11. Soderberg, S. *et al.*, Leptin, but not adiponectin, predicts stroke in males. *J Intern Med* **256** (2), 128-136 (2004).
12. Matsumoto, M., Ishikawa, S., & Kajii, E., Association of adiponectin with cerebrovascular disease: a nested case-control study. *Stroke* **39** (2), 323-328 (2008).
13. Stott, D.J. *et al.*, Adipocytokines and risk of stroke in older people: a nested case-control study. *Int J Epidemiol* **38** (1), 253-261 (2009).
14. Ogorodnikova, A.D. *et al.*, High-molecular-weight adiponectin and incident ischemic stroke in postmenopausal women: a Women's Health Initiative Study. *Stroke* **41** (7), 1376-1381 (2010).
15. Khalili, P. *et al.*, Total adiponectin does not predict cardiovascular events in middle-aged men in a prospective, long-term follow-up study. *Diabetes Metab* **36** (2), 137-143 (2010).
16. Rajpathak, S.N. *et al.*, Resistin, but not adiponectin and leptin, is associated with the risk of ischemic stroke among postmenopausal women: results from the Women's Health Initiative. *Stroke* **42** (7), 1813-1820 (2011).
17. Prugger, C. *et al.*, Adipocytokines and the risk of ischemic stroke: the PRIME Study. *Ann Neurol* **71** (4), 478-486 (2012).
18. Wannamethee, S.G. *et al.*, Adiposity, adipokines, and risk of incident stroke in older men. *Stroke* **44** (1), 3-8 (2013).
19. Gardener, H. *et al.*, Adiponectin and risk of vascular events in the Northern Manhattan study. *Atherosclerosis* **226** (2), 483-489 (2013).
20. Bidulescu, A. *et al.*, Associations of adiponectin and leptin with incident coronary heart disease and ischemic stroke in african americans: the jackson heart study. *Front Public Health* **1**, 16 (2013).
21. Kizer, J.R. *et al.*, Total and high-molecular-weight adiponectin and risk of coronary heart disease and ischemic stroke in older adults. *J Clin Endocrinol Metab* **98** (1), 255-263 (2013).
22. Arregui, M. *et al.*, Adiponectin and risk of stroke: prospective study and meta-analysis. *Stroke* **45** (1), 10-17 (2014).
